# THE INFLUENCE OF TREATMENT DELAY INCREASE RISKS OF CANCER MORTALITY AMONG OLDER ADULTS: A POPULATION-BASED STUDY

**DOI:** 10.1093/geroni/igac059.2684

**Published:** 2022-12-20

**Authors:** Jansen Cambia, Edmund Cedric Orlina, Jason Jiashuan Liu

**Affiliations:** National Yang Ming Chiao Tung University, National Yang Ming Chiao Tung University, Taipei, Taiwan (Republic of China); Department of Health - Rizal Cancer Registry, Department of Health - Rizal Cancer Registry, Rizal, Philippines; National Yang Ming Chiao Tung University, National Yang Ming Chiao Tung University, Taipei, Taiwan (Republic of China)

## Abstract

Treatment delays during the ongoing COVID-19 pandemic has worsened oncologic outcomes, such as increasing early mortality among older adults. We investigated the association of treatment delay on the 2-year and 5-year risk of all-cause mortality, and stratified among age ≤60 and age >60 years. This was a retrospective study of cancer patients using the Department of Health-Rizal Cancer Registry (DOH-RCR) from 1971–2012. We employed Poisson regression analysis to compare the risk of death among patients with different treatment delay lengths, defined as interval from diagnosis to treatment of ≤30 days, 31–60 days, and >60 days. We included 16,472 cancer patients. After adjusting for confounding, the 2-year risk of death was significantly higher among patient with treatment delay of >60 days by IRR=1.27 [95% CI=1.2–1.3] and 5-year risk by IRR=1.21 [95% CI=1.2–1.3], compared to delay of less than 60 days. Consistent findings were observed by age-groups, revealing that delay of >60 days puts age≤60 and age>60 years at higher risk of 5-year mortality, by IRR=1.21 [95% CI=1.2–1.3] and IRR=1.20 [95% CI=1.1–1.3], respectively. Treatment delay was associated with overall cancer mortality for cancer diagnoses at any age range. However, further investigation is needed to understand the predictors of longer treatment delay, which may provide consolation to balanced care during the current pandemic.

